# Factors associated with the recurrence of cholera outbreaks in Rumonge Health District in Southern Burundi: An unmatched case-control study

**DOI:** 10.1371/journal.pone.0354969

**Published:** 2026-08-03

**Authors:** Emile Nkurunziza, Robert Kogi

**Affiliations:** 1 National Institute for Public Health (INSP), Bujumbura, Burundi; 2 Ghana Health Service, Ahafo Regional Health Directorate, Hwidiem; 3 Department of Social and Behavioural sciences, School of Public Health, College of Health Sciences, University of Ghana, Legon, Accra; Cranfield University, UNITED KINGDOM OF GREAT BRITAIN AND NORTHERN IRELAND

## Abstract

Cholera remains a public health concern in Rumonge Health District. The area is regularly affected by the disease outbreaks despite control measures that are periodically taken after the outbreaks occur. The last outbreak started in late 2018 and ended in February 2019, with a cumulative of 234 cases, and one death. This study aimed to identify the risk factors associated with the 2019 cholera outbreak in Rumonge Health District. We conducted a retrospective unmatched (1:2) case-control study to investigate risk factors associated with the 2019 cholera outbreak. This was carried out from 5 March 2021–30 April 2021 in 150 households, comprising 50 cases and 100 controls. Cholera cases were drawn from the admission register of cholera patients at Rumonge referral hospital. Stratified proportional allocation sampling followed by simple random selection were used to select the cases. Afterwards, we visited the households of the selected cases and controls that were chosen among the neighborhood of the cases. An interviewer-administered questionnaire was administered to the head of each household to collect sociodemographic, water, sanitation, and hygiene practice data. Multivariate analysis with logistic regression model was used to analyze the data. A multivariate analysis revealed that drinking lake/river water (OR=3.6 [1.46–8.87]), utilizing untreated water for domestic purposes (OR=5.2 [1.31–20.59]), consuming unwashed raw fruit (OR=2.9 [1.08–7.97]), eating without washing hands with soap (OR=3.2 [1.30–8.29]) and improper washing of hands after defecation (OR=2.7 [1.10–6.84]) were predictors of cholera outbreaks in Rumonge Health District. The study revealed that drinking lake or river water, using untreated water for domestic purposes, consuming unwashed raw fruits, eating without washing hands with soap, and improper handwashing after defecation were all strongly associated with increased risk of cholera. We recommend a community-based approach through the provision of safe water, strengthened hygiene education, and integrated WASH interventions in Rumonge Health District.

## Introduction

Cholera is a diarrheal disease that leads to rapid dehydration, which can be fatal in less than a few hours [1–2]. The World Health Organization (WHO) estimates that 2.8 million people are globally affected by cholera with 9,100 deaths every year [3]. The African continent accounts for over 90% of cholera cases reported to the WHO, and the disease is endemic in 47 sub-Saharan African countries [4–12].

The seventh cholera pandemic emerged in Eastern Africa region in 1978, and from then, several countries have experienced recurrent cholera outbreaks in this area, including Somalia, Democratic Republic of Congo (DRC), Tanzania, Zambia, Uganda, South Sudan and Kenya [13]. Since 1977–1978, Burundi, Rwanda, Democratic Republic of Congo, Tanzania, Uganda, and Kenya have reported cholera cases almost every year [14]. Moreover, since 1990, Burundi, Democratic republic of Congo, and Tanzania, located partly in Great Lake Region that comprise Tanganyika, Kivu, and Victoria lakes record cholera cases almost every year [14].

In Burundi, Imbo area that shares border with DRC by Tanganyika Lake, is among the most affected by cholera outbreaks. Rumonge commune, which is on Lake Tanganyika shore, southern Burundi, was the first to report cholera cases in 1978 [15]. This commune was also affected by cholera outbreak in 1992, with 318 cases and fatality rate of 3% [15]. A study carried out in Burundi in 2021 showed that cholera outbreaks recur almost every year [16]. The recurrence raises questions about the effectiveness of cholera control measures that are regularly taken each time the outbreak occurs**.** To mitigate the negative impact of the cholera outbreak, various public health measures are taken to limit the spread of the disease whenever it occurs. However, these measures, including banning road-side sale of raw vegetables, drinking of Tanganyika Lake, and river water if not treated**,** supplying clean water by tanker trucks, and distributing chlorine tablets to treat water are only for short periods.

Cholera outbreak disrupts socio-economic life of the affected population, and threatens human life [17]. In the event of cholera outbreak, trading and fishing activities on Lake Tanganyika are disrupted and slowed down, and the movement of goods and people is curtailed.

Despite efforts rolled out to manage cholera outbreak, it remains a public health concern in Burundi over the past decades. This recurrence of cholera outbreaks raises concerns about the factors that cause its reappearance. Therefore, this study was conducted to identify the risk factors associated with the 2019 cholera outbreak in Rumonge Health District.

## Materials and methods

### Study setting

Rumonge Health District is situated in the southern part of Burundi, an area bordering the DRC by Lake Tanganyika. The area has very active cross-bordering trade between Burundi, the South Kivu province of DRC, Kigoma, and Katavi provinces of the Republic of Tanzania through the port of Rumonge. Measures to prevent and control infections and surveillance at entry points are not rigorous, which can lead to the emergence of cross-border diseases between these three countries. In addition, fishing activities are active all along the shores of Lake Tanganyika. This fishing activity involves various categories of people, including fishermen, fish buyers, and sellers of various items in small markets set up on the shores of the Lake. Despite this influx of people, especially during the fishing season, sanitary facilities such as drinking water taps, toilets and waste bins are not always available, thereby increasing the risk of outbreaks.

During the fishing season, there are large movements of people and fishing boats, facilitated by the road linking the capital Bujumbura, to Tanzania, and DRC countries.

Moreover, this area is covered by mountains with enormous quantity of water, but the population often faces a shortage of clean water. Some places have no clean water at all, while others have taps that ran dry several years ago. Hence, in Rumonge city, clean water is not always available. Schools, markets, health centers and other places where people meet do not have permanent access to potable water. Even in some areas where it is available, there are frequent water cuts. In addition, the practice of renting water taps to third parties deprive people of accessing potable water as they charge money before one can access it.

### Study design

We conducted a retrospective unmatched case-control study to investigate risk factors associated with the 2019 cholera outbreak. To control for confounding in this design, both design- and analysis-based approaches were employed. Controls were selected from the same neighborhoods as cases to enhance comparability and minimize environmental and socio-demographic differences. During analysis, variables with p-values <0.25 in univariate analysis, along with epidemiologically relevant factors, were included in the multivariable logistic regression model. Adjusted odds ratios were estimated to account for potential confounding, and backward stepwise selection was used to retain variables with statistical significance at p < 0.05.

We developed and administered a questionnaire to the study respondents. This questionnaire consisted of questions including demographic characteristics, households’ hygiene practices, water provision and storage, personal hygiene practices, and sanitation.

### Study participants

Participants were household heads comprising of males and females, regarded as the decision-maker within the household. In the absence of the head of the household, any other person over 18 years of age was eligible to respond to the questionnaire.

### Sampling calculation

The sample size was calculated using OpenEpi software, with a 95% confidence level, a case-control ratio of 1:2, and a test power of 80%. The hypothetical proportion of controls with exposure was set at 50% [18]. Based on these parameters, the final sample consisted of 150 households, including 50 case households and 100 control households.

### Sampling strategy

Any household located in Rumonge Health District area that had a cholera case during the 2019 cholera outbreak was considered as a case. Control household was defined as any household located in Rumonge Health District area that didn’t have any cholera case during the 2019 cholera outbreak.

We set a database of all households that had at least one cholera case during the 2019 cholera outbreak, from the admission register of cholera patients admitted at the cholera treatment center at Rumonge referral hospital. This database included full nouns of households, telephone numbers, and their addresses. Afterwards, we divided Rumonge Health District area into nine administrative zones, and then proportionally allocated household cases per zone. Within each zone, we randomly chose case households. The localization of households was facilitated by a local administrative officer, after showing him the list of households we wanted to visit.

The first household visited was the one close to the local administrative office. Afterwards, we visited the following households until we reached the sample size. The control households were found close to each case household. The data collected included socio-demographic characteristics; water; sanitation and hygiene. To ensure confidentiality, identifiable information obtained from hospital records (including names, telephone numbers, and addresses) were used exclusively for tracing selected households and was not included in the final dataset. Unique identification codes were assigned to each respondent, and all interviews were conducted in a private setting. Access to identifiable data was restricted to the research team, and all data were anonymized prior to analysis to prevent disclosure of individual records.

### Data analysis

We cleaned the data by using Excel and performed analysis using Stata 15.1. We described socio-demographic, hygiene practice characteristics by calculating frequencies. Multivariate logistic regression model was used. Exposure-related variables which were included in the unconditional logistic regression analysis were used in the multivariate model if they displayed a p-value <0.25 in the univariate analysis. From the variables showing p-values <0.25 in the univariate analysis, a subset judged to be epidemiologically meaningful and strongly associated with cholera transmission—drinking river/lake water, decontaminating lake/river water before use, washing raw fruit before consumption, washing hands with soap before eating, and washing hands with soap after defecation were selected for inclusion in the multivariable logistic regression model [19–23]. We calculated the odds ratios (ORs) and 95% confidence intervals (CIs) associated with the independent variables. Model building included backward stepwise selection with an inclusion level of 0.05. Variables with sparse categories data were retained to preserve epidemiological relevance, but their estimates were interpreted cautiously due to potential instability

### Ethical consideration

Ethical approval for this study was obtained from the National Institute for Public Health (INSP) Ethics Committee (IRB approval number: DECISION CIE/04/2021). Detailed information about the study objectives, procedures, risks, and benefits were provided to all participants in a language they understood, prior to data collection.

Informed consent was obtained from each respondent verbally. Following agreement to participate, the interviewers documented the respondent’s verbal consent in a designated section of the questionnaire and recorded the date of consent. The consent documentation procedure was reviewed and approved by the INSP Ethics Committee.

## Results

### Sociodemographic characteristics

The socio-demographic characteristics of respondents in Table 1 showed that the majority of both cases (76%) and controls (76%) resided in rural areas. Among cases, 54% were household heads and 46% were spouses, which was comparable to controls (51% and 49%, respectively). The sex distribution was similar across groups, with males comprising 52% of cases and females accounting for 52%. Most respondents were aged ≥30 years (86% of cases vs. 83% of controls) and in marital unions (78% vs. 86%). Regarding education, illiteracy was more common among cases (32%) than controls (28%), while a higher proportion of cases had only primary education (30%) compared to controls (39%). Household size greater than five people was more frequent among cases (74%) than controls (63%).

**Table 1:** Socio-demographic characteristics of respondents in Rumonge Health District, Burundi, 2019.

Risk factors	Case, n (%)	Control, n (%)
**Residence**		
Urban	12 (24%)	24(24%)
Rural	38 (76%)	76(76%)
**Respondent**		
Head of household	27(54%)	51(51%)
Spouse	23(46%)	49(49%)
**Sex**		
Male	26(52%)	48(48%)
Female	24(48%)	52(52%)
**Age (Years)**		
<30	7(14%)	17(17%)
≥30	43(86%)	83(83%)
**Marital status**		
Union	39(78%)	86(86%)
Non-union	11(22%)	14(14%)
**Education level**		
Illiterate	16(32%)	28(28%)
Literate	34(68%)	72(72%)
**Persons per household**		
≤5	13(26%)	37(37%)
>5	37(74%)	63(63%)

### Factors associated with the recurrence of cholera outbreaks in Rumonge Health District

From Table 2, water provision and storage practices revealed several differences between cases and controls. Awareness of cholera was high across both groups, with 76% of cases and 74% of controls reporting prior information. However, the use of unsafe water sources was more common among cases, with 82% of cases versus 56% of controls reported using lake or river water for domestic purposes, and nearly all cases (98%) drank lake or river water compared to 93% of controls. Importantly, decontamination of surface water before use was rarely practiced among cases (6%) compared to controls (22%). Access to safer water sources was limited, with very few respondents owning a household tap (4% of cases; 6% of controls), and most relying on public taps located less than 500 meters from their households (70% of cases; 64% of controls). Interruptions in water supply were common, with supply line failures (38% of cases; 39% of controls) and dry taps (34% of cases; 25% of controls) being the most frequently reported reasons. Almost all households stored their drinking water in cans (96% of cases; 90% of controls).

**Table 2:** Water provision and storage in Rumonge Health District, Burundi, 2019.

Risk factors	Case, n (%)	Control, n (%)
**Information on cholera**		
Yes	38(76%)	74(74%)
No	12(24%)	26(26%)
**Use of river/lake water for domestic purposes**		
Yes	41(82%)	56(56%)
No	9(18%)	44(44%)
**Drinking lake/river water**		
Yes	49(98%)	93(93%)
No	1(2%)	7(7%)
**Decontamination of lake/river water before their use** ^*^		
Yes	3(6%)	22(22%)
No	47(94%)	78(78%)
**Possession of own tap**		
Yes	2(4%)	6(6%)
No	48(96%)	94(94%)
**Tap functioning**		
Sometimes	1(50%)	4(67%)
Always	1(50%)	2(33%)
**Reason for intermittent functioning**		
Lack of water	1(50%)	4(100%)
Lack of money for repairs	1(50%)	0(0.0%)
**Distance from household to public tap (meters)**		
< 500	35(70%)	64(64%)
500-1000	4(8%)	14(14%)
1000-2000	3(6%)	4(4%)
≥2000	8(16%)	18(18%)
**Reason of closing the public tap**		
The renter has not paid the tap’s operating costs	6(12%)	12(12%)
Supply line failure	19(38%)	39(39%)
Dry tap	17(34%)	25(25%)
Shut off due to draw down schedule	8(16%)	24(24%)
**Reservoir to store drinking water** ^*^		
Can	48(96%)	90(90%)
Tank without lid	2(4%)	10(10%)

*Means variables with small counts.

Household hygiene and sanitation practices varied between cases and controls (Table 3). Regular handwashing with soap was less common amongst cases, both before preparing food (76% vs. 87%) and before eating (62% vs. 87%). Similarly, fewer cases reported cleaning raw vegetables before consumption (68% vs. 89%). Hand hygiene after defecation was particularly poor among cases, with only 20% practicing handwashing with soap compared to 35% of controls. Eating food sold in public places and consuming locally produced beverages were highly prevalent in both groups (>85%). Waste disposal practices were largely similar, with composting being the most common method (66% of cases; 55% of controls). Almost all households reported having toilets, but nearly all case households (97.8%) used board toilets, while slab toilets were more common among controls (43.4%). Few households had toilets with lids (21.7% of cases; 36.4% of controls). Open defecation was rare, though some households reported defecating directly into lakes or rivers (22% of cases; 21% of controls).

**Table 3:** Household hygiene and sanitation practices in Rumonge Health District, Burundi, 2019.

Risk factors	Case, n (%)	Control, n (%)
**Washed hands with soap before preparing food**		
Yes	38 (76%)	87(87%)
No	12(24%)	13(13%)
**Cleaned raw vegetables before consumption**		
Yes	34(68%)	89(89%)
No	16(32%)	11(11%)
**Washed hands with soap before eating**		
Yes	31(62%)	87(87%)
No	19(38%)	13(13%)
**Ate food sold at public place**		
Yes	43(86%)	94(94%)
No	7(14%)	6(6%)
**Consumed locally produced beverages**		
Yes	43(86%)	85(85%)
No	7(14%)	15(15%)
**Washed hands with soap after defecation**		
Yes	10 (20%)	35(35%)
No	40(80%)	65(65%)
**Household waste disposal site**		
Gutter	1(2%)	4(4%)
Open air	15(30%)	40(40%)
Compost	33(66%)	55(55%)
Garbage bag	1(2%)	1(1%)
**Household with toilet**		
Yes	46(92%)	99(99%)
No	4(8%)	1(1%)
**Type of toilet**		
Board toilet	45(97.8%)	56(56.6%)
Slab toilet	0(0.0%)	43(43.4%)
Seated toilet	1(2.2%)	0(0.0%)
**Toilet with lid**		
Yes	10(21.7%)	36(36.4%)
No	36(78.3%)	63(63.6%)
**Place of human waste disposal**		
Dug hole	14(30.4%)	24(24.2%)
Open air	0(0.0%)	0(0.0%)
Septic tank	1(2.18%)	2(2%)
Dig another toilet when filled	25(54.35%)	58(58.6%)
Toilet never filled	6(13.04%)	15(15.2%)
**Destruction of a toilet by heavy rain**		
Yes	7(15.2%)	12(12.1%)
No	39(84.8%)	87(87.9%)
**Defecation in a lake/river**		
Yes	11(22%)	21(21%)
No	39(78%)	79(79%)

Univariate analysis in Table 4 identified several significant risk factors for cholera in Rumonge Health District during the 2019 outbreak. Individuals who drank lake or river water had more than three-fold higher risk of cholera compared to those who did not (OR = 3.57; 95% CI: 1.57–8.14; *p* = 0.002). Similarly, failure to decontaminate surface water before use was associated with a significantly increased risk of more than 4 times of causing cholera (OR = 4.41; 95% CI: 1.25–15.56; *p* = 0.021). Furthermore, poor hygiene behaviours also emerged as strong predictors, where not cleaning raw fruit before consumption (OR = 3.80; 95% CI: 1.60–9.02; *p* = 0.002) and not washing hands with soap before eating (OR = 4.10; 95% CI: 1.81–9.27; *p* = 0.001) were both significantly associated with increased odds of getting cholera.

**Table 4:** Univariate analysis for selected potential risk factors for cholera outbreak in Rumonge health district, 2019.

Risk factors	Groups		OR (95% CI)	p-value
	case (n)	control (n)		
**Marital status (reference: union)**				
Non-union	11	14	1.73(0.72-4.15)	0.219
**Persons per household (reference: ≤ 5)**				
>5	37	63	1.67(0.78-3.54)	0.180
**Drank river/lake water (reference: No)**				
Yes	41	56	3.57(1.57-8.14)	0.002
**Used lake/river water for domestic** **purposes (reference: No)**				
Yes	49	93	3.68(0.44-30.83)	0.228
**Decontaminated lake/river** **water before consumption (reference: Yes)**				
No	47	78	4.41(1.25-15.56)	0.021
**Reservoir to store drinking water** **(reference: can)**				
Tank without lid	2	10	0.37(0.07-1.78)	0.217
**Toilet ownership (reference: Yes)**				
No	4	1	8.60(0.93-79.18)	0.057
**Washed hands with soap before** **preparing food (reference: Yes)**				
No	12	13	2.11(0.88-5.05)	0.093
**Cleaned raw fruit before consumption (reference: Yes)**				
No	16	11	3.8(1.60-9.02)	0.002
**Washed hands with soap before** **eating (reference: yes)**				
No	19	13	4.10(1.81-9.27)	0.001
**Consumed food sold at public** **places (reference: No)**				
Yes	43	94	0.39(0.12-1.23)	0.110
**Washed hands with soap after** **defecation (reference: Yes)**				
No	40	65	2.15(0.96-4.82)	0.062

Other factors, including larger household size (OR = 1.67; *p* = 0.180), lack of toilet ownership (OR = 8.60; *p* = 0.057), and not washing hands after defecation (OR = 2.15; *p* = 0.062), showed elevated odds ratios but did not reach statistical significance. Conversely, consumption of food sold in public places was not associated with increased cholera risk (OR = 0.39; *p* = 0.110).

The multivariate logistic regression analysis in Table 5 confirmed several independent risk factors for cholera outbreak recurrence in Rumonge Health District. Drinking lake or river water remained 3.6 times more strongly associated with cholera (aOR = 3.6; 95% CI: 1.44–8.87; *p* = 0.005). Similarly, failure to decontaminate surface water before use significantly increased the risk of cholera by approximately 5 times (aOR = 5.2; 95% CI: 1.31–20.59; *p* = 0.019). Poor food and hand hygiene practices were also predictive risk factors, where not washing raw fruit before consumption (aOR = 2.94; 95% CI: 1.08–7.98; *p* = 0.034), not washing hands with soap before eating (aOR = 3.28; 95% CI: 1.30–8.29; *p* = 0.012), and not washing hands with soap after defecation (aOR = 2.74; 95% CI: 1.10–6.84; *p* = 0.030) were each independently associated with increased odds of cholera.

**Table 5:** Logistic regression for selected risk factors associated with cholera outbreak recurrence in Rumonge Health District, Burundi, 2019.

Risk factors	Modalities	OR (IC95%)	P-value	OR adjusted (IC95%)	P-value
Drinking river/lake water	Non	1		1	
	Yes	3.57(1.57-8.14)	0.002	3.6(1.44-8.87)	0.005
Decontamination of lake/river water before their use	Yes	1		1	
	Non	4.41(1.25-15.56)	0.021	5.2(1.31-20.59)	0.019
Washed raw fruit before consumption	Yes	1		1	
	Non	3.80(1.60-9.02)	0.002	2.94 (1.08-7.98)	0.034
Washed hands with soap before eating	Yes	1		1	
	Non	4.10(1.81-9.27)	0.001	3.28(1.30-8.29)	0.012
Washed hands with soap after defecation	Yes	1		1	
	Non	2.15(0.96-4.82)	0.062	2.74(1.10-6.84)	0.030

## Discussion

Cholera outbreak remains a public health concern in Burundi, as well as an indicator of social inequity. This study examined the factors associated with the recurrence of the 2019 cholera outbreaks in Rumonge Health District, Southern Burundi. The findings demonstrate that poor food hygiene, poor personal hygiene, and the persistent lack of clean water were the strongest predictors of cholera transmission, consistent with the fecal–oral route as the principal pathway of Vibrio cholerae infection. These results largely corroborate those found by various authors throughout Sub-Saharan Africa [24–26].

In Rumonge Health District, households commonly rely on untreated water from rivers and lakes, reflecting broader structural challenges in access to safe water. This finding is consistent with the well-established fecal–oral transmission pathway of cholera, whereby ingestion of contaminated water or food facilitates the spread of *Vibrio cholerae* [5–8]. The district’s location along Lake Tanganyika, coupled with multiple river systems and limited access to potable water, creates an environment conducive to sustained transmission, particularly during periods of water scarcity. In this study, drinking untreated lake or river water remained a strong independent predictor of cholera, even after adjusting for potential confounders. This result aligns with studies conducted in the Democratic Republic of Congo and Tanzania, where reliance on untreated surface water was strongly linked to recurrent outbreaks [9–10]. Importantly, our findings also reinforce the broader understanding that cholera transmission is not limited to direct consumption of contaminated drinking water [23]. Poor hygiene practices such as inadequate handwashing and consumption of unwashed raw foods highlight the role of non-drinking water pathways in sustaining transmission. These indirect routes, even though often overlooked, are critical components of the fecal–oral cycle and underscore the need for comprehensive interventions that address water quality, food hygiene, and sanitation simultaneously. Moreover, people living near Tanganyika Lake wash their kitchen utensils, clothes, and often had their bath in the lake, sometimes defecating in it, thus polluting it further.

The high dependence on natural water sources observed in this study may be related to limited access to reliable piped water, intermittent functioning of public taps, and the cost barriers associated with private water connections in the study area. However, these interpretations should be made with caution given the study’s relatively small sample size and its focus on a specific outbreak period, which may not fully reflect routine conditions. In addition, the reliance on self-reported data introduces the possibility of recall and social desirability bias, particularly for water access and hygiene practices.

Despite these limitations, the findings suggest that constrained access to safe and reliable water sources may contribute to increased reliance on untreated surface water. This underscores the potential importance of improving equitable access to safe water as part of broader cholera prevention efforts, although further studies with larger sample sizes and more robust designs are needed to confirm these relationships.

Our findings point to unsafe water use for domestic activities and unsafe water storage as the strongest risk factors for cholera infection in this setting. While drinking lake or river water was common across both groups, using untreated surface water for domestic purposes, such as washing utensils, produce, or hands. This was significantly associated with illness (OR ≈3.6). Likewise, households storing drinking water in uncovered tanks had substantially higher odds of being cases compared to those using closed containers (OR≈4.4). These results suggest that transmission is likely occurring through secondary contamination pathways within the household rather than through drinking water alone.

Hygiene behaviours, particularly handwashing with soap before food preparation and eating, also showed strong protective trends, although some estimates were imprecise due to small numbers. Sanitation factors such as toilet presence and type showed mixed crude associations, but earlier analyses indicate that higher-quality sanitation (e.g., slab toilets, lids) is likely protective and may reflect broader socioeconomic or structural advantages. Overall, our data consistently highlight that the key leverage points for reducing cholera risk are ensuring safe domestic water practices, improving water storage, and strengthening critical hygiene behaviours, especially around food preparation and eating.

Furthermore, it was noted in this study that not washing hands with soap before meals, and inadequate handwashing after defecation were also independently associated with cholera outbreak. Our findings specifically revealed that improper handwashing before eating has three times increased risk of cholera than those who properly washed their hands. This could be as a result that in many households, people do not wash their hands with soap and running water, increasing their risk of ingesting vibrio cholerae. Our findings highlighted the importance of hygiene measures during cholera outbreak, as revealed by previous studies carried out across Africa [24,27].

In this study, handwashing with soap after defecation was suboptimal among both cases and controls, suggesting gaps in consistent hygiene practices within the study population. However, this observation was interpreted cautiously, given the limited sample size, the outbreak-specific context, and the reliance on self-reported behaviours, which may be subject to recall and social desirability bias. Rather than contradicting established public health recommendations, these findings highlighted potential challenges in the consistent adoption and sustainability of recommended hygiene practices in resource-constrained settings [28–32].

Furthermore, while consumption of food from public places was not significantly associated with cholera in this study, this does not preclude its role in transmission in other settings. Instead, the findings point to the possible prominence of household-level exposure pathways, including food handling, water storage, and hand hygiene within domestic environments. This suggests that transmission differences may be highly context-specific, with intra-household practices playing a critical role during outbreaks. It also underscores the need to better understand behavioural consistency, access constraints, and micro-environmental factors that influence hygiene practices beyond knowledge alone.

The implications of these findings are multifaceted and consistent with established fecal–oral transmission pathways of cholera. Structural determinants such as inadequate water infrastructure and poverty-driven reliance on unsafe surface water sources reflect well-documented global drivers of cholera, including unsafe water, poor sanitation, and limited access to hygiene resources. At the same time, behavioural factors, particularly inadequate handwashing and poor food hygiene likely amplified transmission within households during outbreaks.

Importantly, these findings also reinforce the role of non-drinking water pathways, which are sometimes underemphasized in many settings including the Burundian context. Practices such as improper hand hygiene, handling of contaminated food, and unsafe domestic water use may contribute substantially to transmission beyond direct consumption of contaminated drinking water. The convergence of these structural and behavioural factors highlights the need for comprehensive interventions that address both water access and everyday hygiene practices to effectively reduce the recurrence of cholera outbreaks in Rumonge.

### Strength and limitations

One limitation of this study is the potential for recall bias arising from the selection of control participants and the retrospective collection of exposure information. Participants may not have recalled previous exposures with complete accuracy, which could have affected the validity of the responses. Also, social desirability bias may have influenced self-reported practices such as handwashing or food hygiene, possibly leading to underreporting of poor behaviours. In addition, the study was conducted in a single health district, which limited the generalizability of the findings to other regions of Burundi with different socio-ecological contexts.

Additionally, although some proxy indicators such as education and household size were included, comprehensive measures of socio-economic status like income or wealth index were not assessed. Also, even though distance to water source was assessed, it was not included in the final multivariable model. These variables could act as confounders, as they may influence both water access and exposure to unsafe water sources. Furthermore, some variables had small sample sizes in certain categories, which may have reduced the precision of the estimated associations and were interpreted with caution. The cross-sectional nature of data collection restricts the ability to infer temporality, although the associations identified remain consistent with established cholera transmission pathways. Other limitations such as household crowding, environmental conditions, and other unmeasured factors may have influenced both exposures and disease status. Also, the study was conducted during an ongoing outbreak, so findings may not be fully generalizable to other settings or non‑outbreak periods.

Despite these limitations, standardized data collection tools, interviewer training, and uniform interviewing procedures for both cases and controls were employed to reduce differential recall bias. This study therefore provided context-specific evidence on the factors sustaining cholera recurrence in Rumonge Health District, with important implications for both local and national cholera prevention strategies.

### Conclusion

This case–control study demonstrates that unsafe water use, inadequate water handling, and poor hygiene practices are key drivers of cholera infection in Rumonge Health District. Behaviours such as drinking and using untreated surface water for domestic tasks, insufficient handwashing with soap, particularly before eating, and inadequate cleaning of raw fruits and vegetables were strongly associated with increased risk. These findings reinforce the central role of water, sanitation, and hygiene (WASH) in interrupting cholera transmission and highlight the need for integrated, community‑level prevention strategies.

From a policy standpoint, the results underscore the urgency of strengthening safe water access not only in settlement settings but in most poor areas in Burundi, especially through reliable, continuously treated piped water. Improving the performance and maintenance of the water supply system, particularly through stronger coordination between local administration and Regideso, will be critical to reducing household dependence on contaminated surface water. In parallel, sustained investments in behaviour‑change programs, affordable chlorine supplies, safe water storage containers, and targeted hygiene promotion are essential to reinforce protective practices at household level.

Given the recurring nature of cholera in the great lake region, a multisectoral approach combining infrastructure improvements, hygiene behavior reinforcement, and rapid community‑based surveillance is required to prevent future outbreaks. Implementing these measures will not only reduce cholera incidence but also contribute to broader public health resilience in Rumonge and across Southern Burundi.

## References

[1] World Health Organization WHO. Cholera vaccines: WHO position paper–August 2017. Weekly Epidemiol Rec. 2017;92(34):477–98. 28845659

[2] Clemens JD, Nair GB, Ahmed T, Qadri F, Holmgren J. Cholera. The Lancet. 2017;390(10101):1539–49. doi: 10.1016/S0140-6736(17)30559-7 28302312

[3] Ali M, Nelson AR, Lopez AL, Sack DA. Updated global burden of cholera in endemic countries. PLoS Negl Trop Dis. 2015;9(6):e0003832. doi: 10.1371/journal.pntd.0003832 PMC4455997 26043000

[4] Mazibuko Z. Epidemics and the health of African nations. Johannesburg: Jacana Media; 2019.

[5] Deen J, Mengel MA, Clemens JD. Epidemiology of cholera. Vaccine. 2020;:1–9. doi: 10.1016/j.vaccine.2019.07.078 31395455

[6] Abubakar A, Bwire G, Azman AS, Bouhenia M, Deng LL, Wamala JF, et al. Cholera epidemic in South Sudan and Uganda and need for international collaboration in cholera control. Emerg Infect Dis. 2018;24(5):883–7. doi: 10.3201/eid2405.171651 29664387 PMC5938777

[7] Oyugi EO, et al. Supplement article an outbreak of cholera in western Kenya, 2015: a case control study. Pan Afr Med J. 2017;28(Supp 1):3–7. doi: 10.11604/pamj.supp.2017.28.1.9477 PMC6113693 30167037

[8] Hounmanou YMG, Mølbak K, Kähler J, Mdegela RH, Olsen JE, Dalsgaard A. Cholera hotspots and surveillance constraints contributing to recurrent epidemics in Tanzania. BMC Res Notes. 2019;12(1):1–6. doi: 10.1186/s13104-019-4731-0 31639037 PMC6805412

[9] Phiri P, Nzala SH, Baboo KS. Factors associated with the recurring cholera outbreaks in Sinazongwe district of southern Zambia. Med J Zambia. 2015;42(4):184–92.

[10] Lubogo M, et al. Oral cholera vaccination coverage in an acute emergency setting in Somalia, 2017. Vaccine. 2020;38(October 2017):A141–7. doi: 10.1016/j.vaccine.2020.01.015 31980193

[11] Mudekereza N, Sia D, Lubambo G, Karemere H. Politique sanitaire, acteurs, stratégies et contenu des interventions de lutte contre le choléra dans la zone de santé d’Uvira en République Démocratique du Congo. Rev Infirm Congo. 2019;3(1):1–9.

[12] Bwire G, Malimbo M, Maskery B, Kim YE, Mogasale V, Levin A. The burden of cholera in Uganda. PLoS Neglected Trop Dis. 2013;7(12):1–10. doi: 10.1371/journal.pntd.0002545 PMC3855006 24340106

[13] Mutreja A, Kim DW, Thomson NR, Connor TR, Lee JH, Kariuki S, et al. Evidence for several waves of global transmission in the seventh cholera pandemic. Nature. 2011;477(7365):462–5. doi: 10.1038/nature10392 21866102 PMC3736323

[14] Nkoko DB, et al. Dynamics of cholera outbreaks in Great Lakes region of Africa, 1978–2008. 10.3201/eid1711.110170 PMC3310557 22099090

[15] Birmingham ME, Lee LA, Ndayimirije N, Nkurikiye S, Hersh BS, Wells JG, et al. Epidemic cholera in Burundi: patterns of transmission in the Great Rift Valley Lake region. Lancet. 1997;349(9057):981–5. doi: 10.1016/S0140-6736(96)08478-4 9100624

[16] Debes AK, et al. Cholera hot-spots and contextual factors in Burundi, planning for elimination. Trop Med Infect Dis. 2021;76(6):1–12. doi: 10.3390/tropicalmed6020076 PMC8163194 34064986

[17] Mogasale V, Ngogoyo SM, Mogasale VV. Model-based estimation of the economic burden of cholera in Africa. BMJ Open. 2021.doi: 10.1136/bmjopen-2020-044615 PMC7993295 33757949

[18] SSCohort.htm. Accessed 2025 August 5. https://www.openepi.com/SampleSize/SSCohort.htm

[19] Cholera Management Guidelines Final 20.11.2023.

[20] Asantewaa. Cholera outbreaks in low- and middle-income countries in the last decade a systematic review and me. 2024. 10.3390/microorganisms12122504 PMC11728267 39770707

[21] Challa JM, Getachew T, Debella A, Merid M, Atnafe G, Eyeberu A, et al. Inadequate hand washing, lack of clean drinking water and latrines as major determinants of cholera outbreak in Somali Region, Ethiopia in 2019. Front Public Health. 2022;10:845057. doi: 10.3389/fpubh.2022.845057 35602140 PMC9120658

[22] Understanding cholera dynamics in African countries with persistent outbreaks: a mathematical modeling approach. BMC Public Health. 10.1186/s12889-025-24507-0 PMC12506165 41063102

[23] Nguyen TT, Kim C, Goucher G, Kim J-H. Associations of water quality with cholera in case-control studies: a systematic review and meta-analysis. BMC Infect Dis. 2025;25(1):1165. doi: 10.1186/s12879-025-11533-x 41013364 PMC12465603

[24] Bompangue D, et al. Lakes as source of cholera outbreaks, Democratic Republic of Congo. Emerg Infect Dis. 2008;14(5):798–800. doi: 10.3201/eid1405.071260 18439365 PMC2600234

[25] Dan-Nwafor CC, et al. A cholera outbreak in a rural north central Nigerian community: an unmatched case-control study. BMC Public Health. 2019;19(1):1–7. doi: 10.1186/s12889-018-6299-3 30683078 PMC6347749

[26] Monje F, et al. A prolonged cholera outbreak caused by drinking contaminated stream water, Kyangwali refugee settlement, Hoima District, Western Uganda: 2018. Infect Dis Poverty. 2020;9(1):1–10. doi: 10.1186/s40249-020-00761-9 33148338 PMC7640409

[27] Otieno E, et al. Knowledge, attitude and practices on cholera in an arid county, Kenya, 2018: a mixed- methods approach. PLoS One. 2020;428:1–14. doi: 10.1371/journal.pone.0229437 PMC7043758 32101587

[28] Matapo B, Chizema E, Hangombe B, Chishimba K, Mwiinde A, Mwanamwalye I, et al. Successful multi-partner response to a cholera outbreak in Lusaka, Zambia 2016: a case control study. mjz. 2016;43(3):116–22. doi: 10.55320/mjz.43.3.322

[29] Dureab F, Jahn A, Krisam J, Dureab A, Zain O, Al-Awlaqi S, et al. Risk factors associated with the recent cholera outbreak in Yemen: a case-control study. Epidemiol Health. 2019;41:e2019015. doi: 10.4178/epih.e2019015 31010279 PMC6533552

[30] Moradi G, Rasouli MA, Mohammadi P, Elahi E, Barati H. A cholera outbreak in Alborz Province, Iran: a matched case-control study. Epidemiol Health. 2016;38:e2016018. doi: 10.4178/epih.e2016018 27188308 PMC4967910

[31] Gidado S, Awosanya E, Haladu S, Ayanleke HB, Idris S, Mamuda I. Cholera outbreak in a naïve rural community in Northern Nigeria: the importance of hand washing with soap. 2018;8688(September 2010):1–7. doi: 10.11604/pamj.2018.30.5.12768 PMC6093587 30123408

[32] Dubois AE, Sinkala M, Kalluri P, Makasa-Chikoya M, Quick RE. Epidemic cholera in urban Zambia: Hand soap and dried fish as protective factors. Epidemiol Infect. 2006;134(6):1226–30. doi: 10.1017/S0950268806006273 16623992 PMC2870514

